# ERYTHRODERMA: REVIEW OF A POTENTIALLY LIFE-THREATENING DERMATOSIS

**DOI:** 10.4103/0019-5154.48976

**Published:** 2009

**Authors:** Cynthia Okoduwa, W C Lambert, R A Schwartz, E Kubeyinje, A Eitokpah, Smeeta Sinha, W Chen

**Affiliations:** *From the Department of Dermatology and Pathology, New Jersey Medical School, 185 South Orange Avenue, Newark, New Jersey 07103, USA*

**Keywords:** *Erythroderma*, *causes*, *African patients*

## Abstract

Erythroderma, or generalized exfoliative dermatitis, is a disease characterized by erythema and scaling of greater than 90% of the body's surface. The resultant dysmetabolism is potentially life threatening. A detailed history is to identify and treat the underlying cause of this dermatitis. We present two cases of erythroderma in African patients and review this important disease.

## Introduction

Erythroderma is an intense generalized redness of the skin; it was first described by Von Hebra in 1868. It is an inflammatory disorder characterized by an extreme state of skin dysmetabolism that gives rise to extensive erythema and scaling all over the body. This condition classically involves greater than 90% of the body surface. The erythrodermic state is of great concern because it poses significant risk of morbidity and mortality, in addition to the risks inherent to the underlying disease and its therapy.

Erythroderma can be fatal, even when properly managed, primarily because of its metabolic burden and complications. Hence it is mandatory to establish its etiopathology in order to facilitate precise management. This disorder may be the morphologic presentation of a variety of cutaneous and systemic diseases, and a thorough workup is essential. A detailed outline of the patient's history to elicit possible triggering events, including infections, drug ingestion, topical application of medications, sun/ultraviolet exposure and other factors, should be determined. Management of the skin disorder continues to be a challenge due to its multiple etiologies. The prognosis of erythroderma is determined by its underlying cause.

There is a paucity of information on erythroderma in Africa. The growing increase in use of herbal medicines,[1] the HIV epidemic,[2] hospital visits in advanced disease stages and limited investigational resources in Third World environments — all exacerbate the life-threatening nature of this condition. With increasing use of herbal and nonherbal medicines, more individuals are at risk of contracting erythroderma.

## Two Illustrative Cases

### Case 1

**Figure 1:**
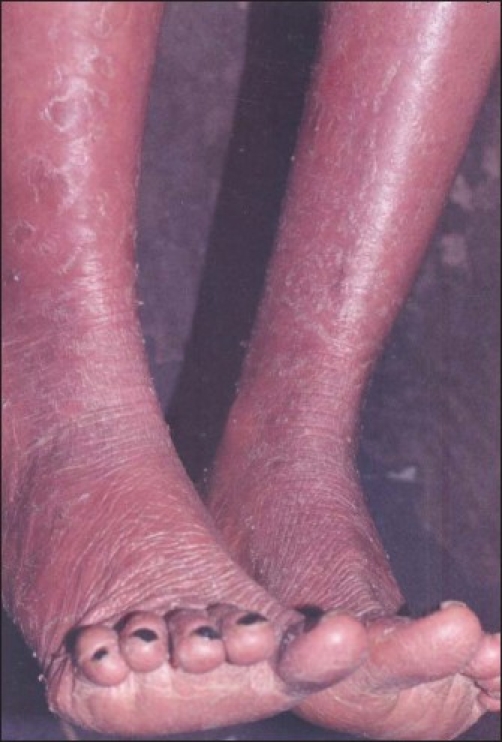
Fifteen-years-old girl with diffuse erythema and scaling of lower extremities

**Figure 2:**
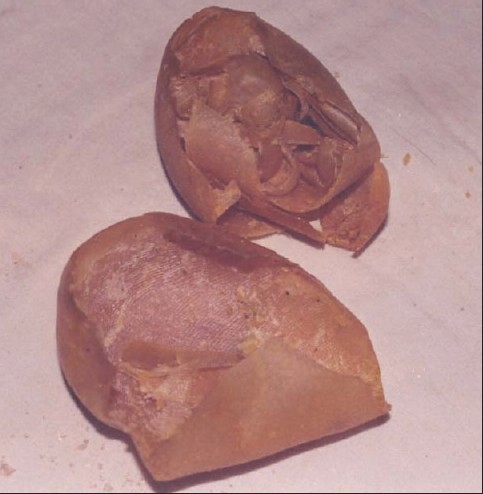
Sloughing of epidermis of the soles in the above patient

A 14-year-old African girl was referred to our Benin City clinic in 2005 for generalized scaling and erythema of 5 years duration. The pruritic patches were first noted on the face and trunk and later involved the entire body. There was no preexisting dermatosis, nor prior medical problems. There was no prior exposure to chemical precipitants of dermatitis. Family history was negative for similar conditions or skin disorders. The exfoliation did not improve with herbal medicines (Yoruba Agbo leaves). Physical examination showed extensive non-uniform erythematous scaly patches involving the scalp, face, trunk, arms, legs, palms and soles [Figure 1]. Scalp lesions formed whitish yellow scales with hair loss. On the soles, the exfoliative eruption led to severe sloughing of the epidermis [Figure 2]. Laboratory investigations and biopsy results were nonspecific. The etiology could not be determined due to limited resources in our clinic. She was treated with intravenous and topical steroids and was lost to follow-up.

### Case 2

**Figure 3:**
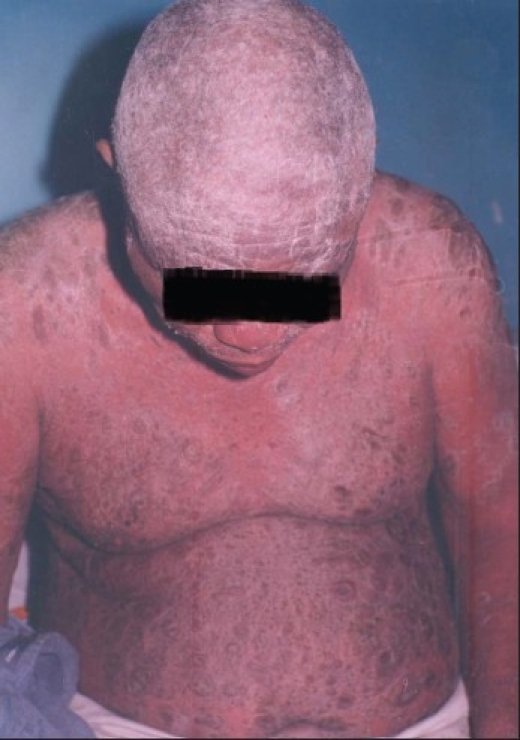
Sixty-four-years-old man with papulosquamous plaques on the scalp, arms and trunk

**Figure 4:**
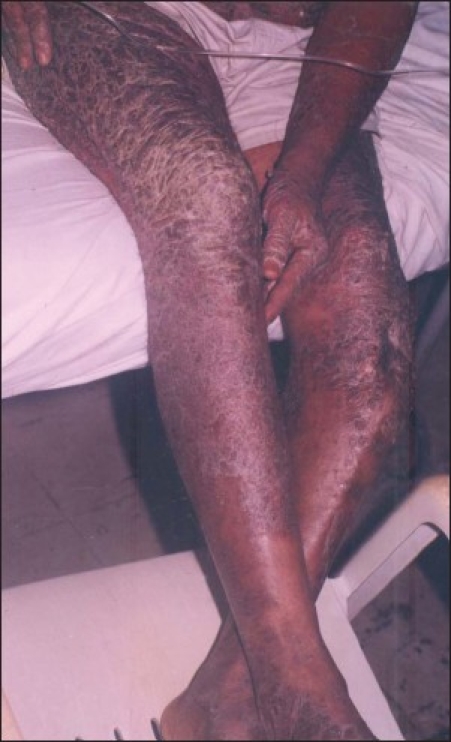
Erythematous scaling plaques of the lower extremities

A 64-year-old man presented to our Benin City clinic in 2005 with an acute eruption of numerous erythematous plaques that had appeared 2 weeks following oral intake of an unknown amount of Aloe vera leaves taken to enhance well-being. There was no history of preexisting dermatologic or medical conditions. Physical examination revealed scaling plaques with prominence on the scalp, trunk and extremities [Figure 3, Figure 4]. Laboratory investigations were consistent with dehydration and inflammatory changes. Topical and oral steroids achieved rapid improvement of the lesions.

## Epidemiology

The true incidence of erythroderma is unknown. Gehgal and Srivastava[3] performed a large prospective study in the Indian subcontinent, where they determined the incidence to be 35 per 100,000 dermatologic outpatients. Sigurdsson[4] recorded the annual incidence in the Netherlands to be 0.9 per 100,000 inhabitants. In general, studies have shown a male predominance, with the male-to-female ratio ranging from 2:1 to 4:1, and the mean age between 40 and 60 years.[3] Rym *et al.* conducted a retrospective study of 80 erythrodermic adults, looking at patients examined between 1981 and 2000.[5] Patient information included clinical, laboratory, histopathological and therapeutic data. The incidence of erythroderma from the study was 0.3%, the average age being 53.78 ± 18 years; and the male-to-female ratio, 2.2:1. These numbers may, however, underestimate the current statistics in Third World environments such as those in Africa.

## Etiology

A major challenge lies in establishing the underlying cause of erythroderma. Most published series reveal that the majority of patients are diagnosed with psoriasis, spongiotic dermatitis, drug reactions or cutaneous T cell lymphoma (CTCL).[6,7]

A preexisting dermatosis is the single most common cause of adult erythroderma.[3,5,8–13] A number of dermatoses can progress to erythroderma, but the most common include psoriasis and eczema.[3,5,9,12] Rym *et al.* reported 41 of 80 erythrodermic patients had psoriasis,[5] a finding not inconsistent with Spanish, Middle Eastern and Indian studies.[3,9,12] Psoriatic erythroderma may occur in relation to withdrawal of systemic or topical glucocorticoids, use of systemic medications such as lithium and antimalarials, phototherapy burns, infection and systemic illnesses.[14]

The apparent increase in the incidence of exfoliative dermatitis may have a bearing on the introduction of many new drugs. It is therefore important to consider all drug exposures.[14,15] Patients presenting with morbilliform, lichenoid or urticarial drug eruptions may develop generalized exfoliative dermatitis.[16] This association has been inferred from descriptions of patients on antiepileptic medications, antihypertensive agents, antibiotics, calcium channel blockers and a variety of topical preparations.[14,17] Severe exfoliative erythrodermic dermatitis has been reported with use of proton pump inhibitors.[18] Morar[19] identified adverse drug reactions to antituberculosis medication as the most common cause of erythroderma in HIV-seropositive South African patients. Rym *et al.*[5] implicated carbamazepine, phenobarbital, penicillin and paracetamol. The intake of herbal medicines, being very popular, may also increase risk among Africans.

Erythroderma may be a cutaneous manifestation of malignancy. The incidence of internal malignancy is approximately 1%.[20] Reticuloendothelial neoplasms, as well as internal visceral/blood vessel malignancies, may manifest as erythroderma.[16,21] Laryngeal, thyroid, lung, esophageal, gallbladder, gastric, colon, fallopian tube and prostate carcinomas and lymphomas have all been implicated.[22–29] Other associations include cutaneous T cell lymphomas, which comprise mycosis fungoides and Sézary syndrome.[20,30–32] Mycosis fungoides may progress from, accompany, or follow T cell lymphomas, and their presentation may be similar to benign erythroderma.[16,30,33,34] The ability to distinguish malignant from benign erythroderma may require an immunophenotypic study with the use of advanced antibody panels.[35] Definitive diagnosis of erythroderma due to internal malignancy cannot be made based on clinical presentation alone, but a concomitant history of insidious development, progressive decompensation, refractoriness to standard therapeutics and absence of prior skin pathology may be existent.[28]

Erythroderma is also associated with disorders that are not readily classified. It has been reported in association with dermatophytosis, hepatitis, renal failure, acquired immunodeficiency syndrome, congenital immunodeficiency syndrome (Omenn syndrome), graft-versus-host disease, histoplasmosis, lupus, dermatomyositis, thyrotoxicosis and sarcoidosis.[36–47]

## Pathogenesis

Currently, the mechanism of erythroderma is unclear. Adhesion molecules and their ligands play a significant role in endothelial-leukocyte interactions, which impact the binding, transmigration and infiltration of lymphocytes and mononuclear cells during inflammation, injury or immunological stimulation.[48] The rise in adhesion molecule expression (VCAM-1, ICAM-1, E-selectin and P-selectin) seen in exfoliative dermatitis stimulates dermal inflammation, which may lead to epidermal proliferation and increased production of inflammatory mediators.[48] The complex interaction of cytokines and cellular adhesion molecules such as interleukin-1, -2 and -8; intercellular adhesion molecule-I (ICAM-I); and tumor necrosis factor (TNF)[49] results in significantly elevated epidermal turnover rate, leading to above-normal mitotic rate.[50] The amount of germinative cells increases and the transit time of keratinocytes through the epidermis decreases, causing loss of more cellular material from the surface.[14]

Sigurdsson *et al.* conducted an immunohistochemical study and observed that the dermal infiltrate in patients with Sézary syndrome mainly showed a T-helper 2 (Th2) cytokine profile, in contrast to a T-helper 1 (Th1) cytokine profile in benign reactive erythroderma, which suggests that a relatively uniform clinical picture in erythroderma does not imply similar pathomechanisms for various etiologies.[48,51]

## Clinical Features

The pattern observed is erythematous patches, which increase in size and coalesce to form extensive areas of erythema, and eventually spread to involve most of the skin surface.[50,52] Some studies have shown sparing of the nose and paranasal areas, and this has been described as the “nose sign”.[53,54]

The epidermis appears thin, giving a glossy appearance to the skin. Once erythema has been established, white or yellow scales develop that progress to give the skin a dry appearance with a dull scarlet and gray hue.[14] Induration and thickening of the skin from edema and lichenification may provoke a sensation of severe skin tightness in the patient.[14] The skin is bright red, dry, scaly and warm to touch.

Some patients may experience involvement of their palms and soles, with hair loss and nail shedding.[55] Involved nails are thick, lusterless, dry, brittle, and show ridging of the nail plate.[50] Subungual hyperkeratosis, distal onycholysis, splinter hemorrhages occur; and sometimes, the nails may shed.[14] Shelley[56] described alternating bands of nail plate discontinuity and leukonychia in drug-induced erythroderma.

Sometimes, the clinical presentation may be suggestive of the underlying cause. Typical psoriasiform plaques may be apparent in early erythrodermic psoriasis. Pityriasis rubra pilaris shows islands of sparing, orange-colored palmoplantar keratoderma and hyperkeratotic follicular papules on juxta-articular extensor surfaces.[14] The violaceous papules and reticulated buccal mucosal lesions of lichen planus may be evident. Joly *et al.*[57] described three African patients presenting with lichenoid erythroderma different from the classic form of lichen planus pemphigoides. The presence of heavy crusts on the palms and soles with subungual hyperkeratosis raises the possibility of Norwegian scabies.[14] In patients with pemphigus foliaceus, crusted patches and erosions may appear on the face and upper trunk. A heliotrope rash, poikiloderma, Gottron's papules, periungual telangiectases and muscle weakness may be seen in erythrodermic dermatomyositis.[26,42] Papuloerythroderma of Ofuji presents in elderly men as flat-topped red papules that become generalized erythrodermic plaques without the involvement of abdominal skin folds (“deck chair” sign).[58–60]

Postoperative erythroderma, a type of graft-versus-host disease following surgery along with blood transfusion, is marked by erythroderma, fever, pancytopenia, hepatic insufficiency and diarrhea and may be fatal.[50] Exfoliative dermatitis may also develop in HIV-infected patients with florid manifestations.[38]

The presence of lymphadenopathy and hepatosplenomegaly, particularly in association with liver dysfunction and fever, may suggest a drug hypersensitivity syndrome or malignancy.[14] Gynecomastia has been reported in some patients, possibly reflecting a hyperestrogenic state, although the significance of this finding is unclear.[13]

## Laboratory Findings

Laboratory findings in the erythrodermic patient are usually nonspecific.[16] Common abnormalities are mild anemia, leukocytosis with eosinophillia, elevated sedimentation rate, decreased serum albumin, increased uric acid, abnormal serum protein electrophoresis with polyelevation in the gamma globulin region and elevated IgE levels.[49,61] Eosinophilia is generally a nonspecific finding, although a highly elevated count raises the possibility of a lymphoma.[52] Circulating Sézary cells may be present; but whereas less than 10% is considered nonspecific in the setting of erythroderma, the presence of 20% or more raises the suspicion for Sézary syndrome.[13,62]

## Histopathology

Biopsy specimens tend to have many nonspecific features.[16] Hyperkeratosis, parakeratosis, acanthosis and a chronic perivascular inflammatory infiltrate, with or without eosinophils, are examples.[6] The clinicopathologic correlation is difficult because nonspecific features of erythroderma may mask the specific features of an underlying dermatosis. Direct immunofluorescence studies may be of diagnostic utility in cases of erythroderma secondary to pemphigus foliaceus, bullous pemphigoid, graft-versus-host disease and connective tissue disorders.[52]

## Management

The initial management of erythroderma is the same regardless of etiology. This should include replacement of nutritional, fluid and electrolyte losses.[6] Local skin-care measures should be employed, such as oatmeal baths as well as wet dressings to weeping or crusted sites followed by the application of bland emollients and low-potency corticosteroids.[63] Known precipitants and irritants are to be avoided; and underlying cause, with its complications, is to be treated.[6,16,50] Secondary infections are treated with antibiotics. Edema in dependent areas, such as in periorbital and pedal areas, may require diuretics.[63] Hemodynamic or metabolic instability should be addressed adequately. Serum protein, electrolyte and blood urea levels should be monitored. This condition may resist therapy until the underlying cause is treated; hence it is important to determine underlying etiology early in its management.[63,64] In Africa and other Third World environments, however, this may not be possible.

## Course

The disease course is greatly influenced by etiology. It is progressive when due to drug allergy, lymphoma, leukemia, contact allergens or staphylococcal scalded skin syndrome.[3,6,13,50] A slower course is observed if from a primary skin disease such as psoriasis or atopic dermatitis.[3,6,13,50] Drug-induced erythroderma patients recover completely with prompt diagnosis and treatment.[6,50] The outcome is unpredictable in idiopathic erythroderma, and its course is marked by multiple exacerbations; prolonged corticosteroid use is often required.[16,50,62]

## Prognosis

Conflicting reports have been published regarding the prognosis of patients with erythroderma in developed nations. The most common causes of death in patients with erythroderma are pneumonia, septicemia and heart failure.[16,63] Elderly patients who develop complications such as infection, fluid/electrolyte imbalances and cardiac failure are at higher risk of mortality.[16,50] Initial studies record death rate in the range of 4.6% to 64%,[5,9,16,49,50,61] but this has since been reduced due to advancement in diagnosis and therapy.[50] In Third World countries, such as parts of Africa, the former prognosis may be more accurate.
